# Effects of Orally Consumed *Rosa damascena* Mill. Hydrosol on Hematology, Clinical Chemistry, Lens Enzymatic Activity, and Lens Pathology in Streptozotocin-Induced Diabetic Rats

**DOI:** 10.3390/molecules24224069

**Published:** 2019-11-10

**Authors:** İlker Demirbolat, Cansu Ekinci, Fadime Nuhoğlu, Murat Kartal, Pelin Yıldız, Melin Özgün Geçer

**Affiliations:** 1 Phytotherapy Research Center, Bezmialem Vakıf University, 34093 Fatih, İstanbul, Turkey; mkartal@bezmialem.edu.tr; 2 Department of Ophthalmology, Faculty of Medicine, Bezmialem Vakıf University, 34093 Fatih, İstanbul, Turkey; cekinci@bezmialem.edu.tr (C.E.); fnuhoglu@bezmialem.edu.tr (F.N.); 3 Department of Pathology, Faculty of Medicine, Bezmialem Vakıf University, 34093 Fatih, İstanbul, Turkey; pyildiz@bezmialem.edu.tr (P.Y.); mgecer@bezmialem.edu.tr (M.Ö.G.)

**Keywords:** *Rosa damascena*, rose hydrosol, diabetes, cataract, hematology, clinical chemistry, lens pathology, aldose reductase

## Abstract

Diabetes mellitus is a multisystemic metabolic disorder that may affect the eyes, kidneys, vessels, and heart. Chronic hyperglycemia causes non-enzymatic glycation of proteins and elevation of the polyol pathway resulting in oxidative stress that damages organs. The current study aimed to investigate the dose-dependent effects of orally consumed *Rosa damascena* Mill. hydrosol on hematology, clinical biochemistry, lens enzymatic activity, and lens pathology in streptozotocin (STZ)-induced diabetic rats. Diabetes was induced into male Sprague–Dawley rats by intraperitoneal administration of STZ (40 mg/kg body weight). Rose hydrosols containing 1515 mg/L and 500 mg/L total volatiles (expressed as citronellol) were introduced to rats orally for 45 days. Consumption of 1515 mg/L volatile containing rose hydrosol successfully ameliorated hematologic, hepatic, and renal functions. Hydrosols also attenuated hyperglycemia and decreased the advanced glycation end-product formation in a dose-dependent manner. Rose hydrosol components significantly increased the lens enzymatic activities of glutathione peroxidase and decreased the activity of aldose reductase to prevent cataractogenesis. Histopathological examinations of rat lenses also indicated that increasing the dose of rose hydrosol had a protective effect on lenses in diabetic conditions. Additionally, in silico modeling of aldose reductase inhibition with rose hydrosol volatiles was carried out for extrapolating the current study to humans. The present results suggest that rose hydrosol exerts significant protective properties in diabetes mellitus and has no toxic effect on all studied systems in healthy test groups.

## 1. Introduction

Diabetes mellitus (DM) is a metabolic disorder characterized by high blood glucose levels owing to impaired insulin secretion or insulin resistance. Due to the fact of chronic hyperglycemia, many complications over different organ systems, such as the eyes, kidneys, heart, blood vessels, and nerves, may occur [1]. In diabetic patients, the major cause of visual loss is considered to be the cataract formation. Both the severity and duration of diabetes affect the incidence and progression of cataracts [2].

While the etiology of cataracts in diabetic patients is not completely understood, the major mechanism responsible for cataract formation is thought to be oxidative stress. High amounts of glucose were found to affect the electron transfer process in mitochondria, resulting in excess production of superoxide ions [3] which causes oxidative stress. High glucose levels also induce oxidative stress via auto oxidation [4] and non-enzymatic glycation [5]. Reactive oxygen species (ROS) could occur during advanced glycation end-products (AGEs) formation [6]. Glycation could also pacify the antioxidant defense system by inactivating antioxidant enzymes [7,8]. Hyperglycemia could also upregulate the polyol pathway to induce oxidative stress via increasing the activity of aldose reductase (AR) which catalyzes the reduction of glucose to sorbitol. Sorbitol further proceeds the osmotic changes in lens fibers to produce sugar cataracts [9,10,11].

Although surgery is accepted to be the only treatment for cataracts, non-surgical approaches, with little or no side effects, are of pharmaceutical interest to reduce and prevent cataract formation and avoid financial load and intra- or postoperative complications. As the polyol pathway is found to be the prime mediator of diabetic-induced oxidative stress in the lens [12], there has been much interest in AR—the first and rate limiting enzyme of the polyol pathway—inhibitors to prevent cataractogenesis. Aldose reductase inhibitors have structurally diverse and different molecules including synthetic small molecules in plant extracts.

Rose is a common name for woody perennial flowering plants of the genus *Rosa*, in the family Rosaceae. More than 100 *Rosa* species are present around the world and 25 of them have been identified in the flora of Turkey. *Rosa damascena* Mill. is a member of rose species cultivated for the production of rose oil and rose hydrosol. It has long been used as traditional herbal medicine in different countries. Akkadian, Assyrian, Roman, and Hittite histories indicate rose and its usage. Avesta (the primary collection of religious texts of Zoroastrianism) describes the religious significance of rose and rose essences. Dioscorides described the effect of rose on the womb, ears, and eyes as a therapeutic plant. Al-Kindi and Al-Dinawari—9th century physicians—both mentioned rose as a pain reliever for ulcer and oral diseases. A physician from the same era, Zakariya Al-Razi, prescribed rose for the alleviation of hangovers after alcohol consumption. Ibn-I Sina and Ibn-Al-Baitar stated the benefits of rose hydrosol and rose fragrances for heart and cognitive functions [13,14]. All known pharmacological effects, including antidiabetic activity over glucosidase inhibition, are well documented in previous studies [15,16].

Rose hydrosol is a byproduct produced during the steam distillation of rose petals. It contains 2-phenylethanol, citronellol, and geraniol as major substituents and other monoterpene alcohols such as alpha terpineol, 4-terpineol, linalool, and nerol. It also contains allyl chain-substituted guaiacol derivatives like eugenol and methyl eugenol [17]. Monoterpenes show antihyperglycemic and anti-inflammatory properties [18,19]. Studies support that citronellol and geraniol have antihyperglycemic activities via ameliorating the hepatic key enzymes of carbohydrate metabolism [20,21]. The hepatoprotective effect of geraniol was previously studied on rats after a partial hepatectomy [22]. Eugenol, on the other hand, has also been reported as an antihyperglycemic agent and AGEs formation inhibitor [23,24]. A mechanistic investigation of the anticholinesterase activity of rose oil components found that 2-phenylethanol showed the highest anticholinesterase effect and was also more selective towards BChE than AChE [25]. Rose hydrosol has been used in Ayurvedic treatment for eye health and, according to Ibn-Al-Baitar, boiling rose water and exposing the patient’s head to its steam can have healing benefits, especially for eye diseases [14].

The current study aimed to investigate the ameliorating effects of orally consumed rose hydrosol on lens enzyme activities in cataract development, hematology, and clinical biochemistry parameters in STZ-induced diabetic rats and the chronic oral toxicity of rose hydrosol itself.

## 2. Results

### 2.1. Quantifying Rose Hydrosol Volatiles

**Figure 1:**
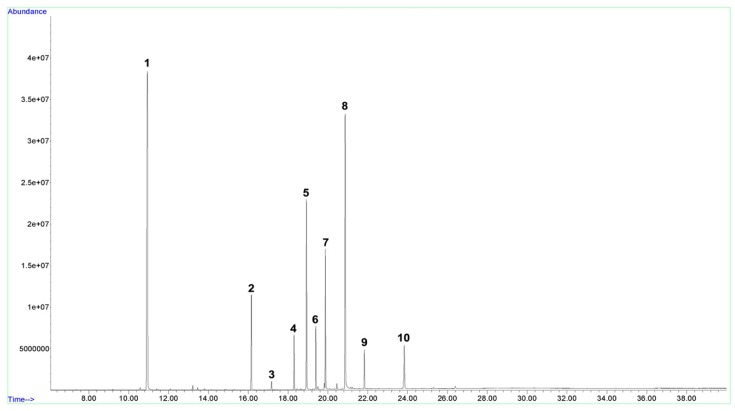
Total ion chromatogram of rose hydrosol. **1**: 1,8-cineole (internal standard), **2**: linalool, **3**: 4-terpineol, **4**: alpha terpineol, **5**: citronellol, **6**: nerol, **7**: geraniol, **8**: 2-phenlyethanol, **9**: methyl eugenol, **10**: eugenol.

First, distillate rose hydrosol was obtained from a local distillery (Isparta, Turkey). Citronellol and 1,8-Cineole reference substances were purchased from Sigma–Aldrich (St. Louis, MO, USA). Various amounts of citronellol solutions were prepared by dissolving them in internal standard solution (0.2% 1,8-Cineole in *n*-hexane) to construct a calibration curve between 1,8-Cineole and citronellol. Forty milliliters of rose hydrosol were oversaturated with sodium chloride and extracted with 10 mL internal standard solution. An Agilent Model 7890B gas chromatograph (CA, USA) equipped with an Agilent 5977E MSD electron impact ionization mass detector was utilized to quantify total monoterpene alcohols expressed as citronellol. Separation was performed on an Agilent HP-Innowax column (60 m, 0.25 mm, 0.25 μm). The injection temperature was maintained at 250 °C, while the helium gas flow was 1.5 mL/min with a split value of 20:1, and a 5 μL sample was introduced to the system. The Aux heater, MS source, and MS quadrupole temperatures were 250 °C, 230 °C, 150 °C, respectively. The scan range was set to 35–200 *m*/*z*. The column temperature program started at 70 °C isothermal for 5 min, then 10 °C/min to 230 °C and stayed for 19 min isothermal at 230 °C with a total of 40 min analysis time. Figure 1 demonstrates the sample chromatogram.

The results of the analysis indicated that the rose hydrosol used in the experiment (RH1) contained 1515 mg/liter total volatiles expressed as citronellol with relative percentages of 8.41% linalool, 0.80% 4-terpineol, 4.87% alpha terpineol, 19.20% citronellol, 5.75% nerol, 13.20% geraniol, 35.98% 2-phenylethanol, 3.70% methyl eugenol, and 6.00% eugenol. Then, RH1 was diluted with tap water to obtain 500 mg/L (RH2) total volatiles.

### 2.2. Water Intakes and Body Weights

**Figure 2:**
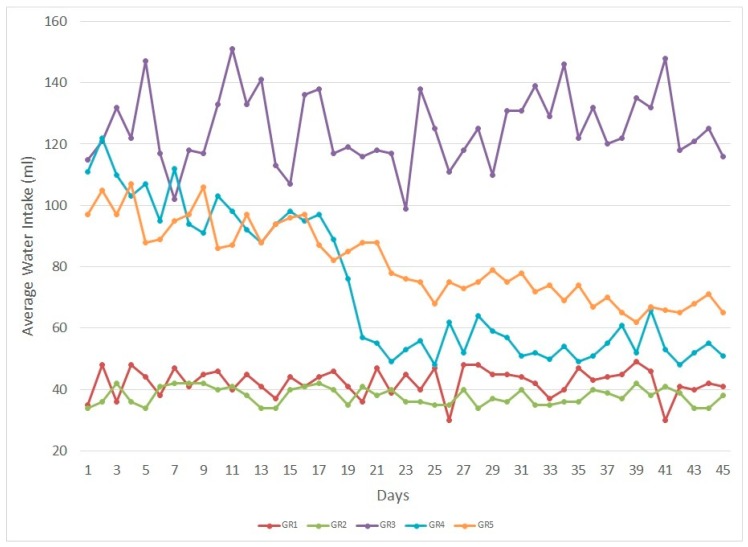
Average water intakes for 45 days.

Water intakes were recorded on a daily basis, and a 45 day average value was used to calculate the rose hydrosol volatiles consumed. Two days after STZ injection, water intake increased in diabetic rats. Overall water consumption of Group (GR)4 and GR5 started to decrease after three weeks in comparison to GR3, and the body weights of GR1 and GR2 increased. The body weights of GR3, GR4, and GR5 decreased as an effect of DM. Table 1 shows the experimental groups, average water intakes, body weights, and average rose hydrosol volatiles (expressed as citronellol) consumption per rat. Figure 2 demonstrates the water intakes for 45 days.

### 2.3. Hematological Parameters

Red blood cell (RBC), hemoglobin (HGB), platelet (PLT), and white blood cell (WBC) counts showed significant changes in GR3 compared to normal and treated rats. The RBC, HGB, and PLT counts decreased. The WBC counts increased as an effect of DM. The prominent differences in the hematologic profiles of the diabetic rat groups drastically normalized with treatment. Both GR1 and GR2 showed no significant differences. The hematological analysis results are shown in Table 2.

### 2.4. Biochemical Parameters

Biochemical parameters were investigated on serum samples. The fasting blood glucose (GLUC) levels of the diabetic groups were significantly elevated in comparison to the normal rats. Post-hoc statistical tests revealed that the GLUC levels of GR4 notably decreased compared with the diabetic groups. As expected, the HbA1c levels were also higher in the diabetic groups and, during the treatment period, the HbA1c levels notably decreased in GR4. The albumin (ALB), bilirubin (BIL), globulin (GLO), and overall serum total protein (TP) levels were higher in diabetic rats but slightly decreased in GR4. Blood urea nitrogen (BUN), urea (UREA), uric acid (UA), and creatinine (CREA) levels notably increased in the diabetic groups. There were significant reductions in all renal function parameters of GR4 in comparison to GR3. The ALT, AST, and ALP levels were higher in the diabetic group as an effect of the STZ injection followed by the induced diabetic condition. Levels of hepatic enzymes decreased noticeably in GR4 throughout the treatment period. Protein glycation and formation of AGEs are common symptoms in diabetes. Diabetic groups have higher levels of AGEs than normal rats, and treatment decreased the levels of AGEs as a result of the decrease in GLUC. The results of all analyses are presented in Table 3. Both GR1 and GR2 showed no significant changes in the post-hoc tests.

### 2.5. Lens enzymatic Activities

Lens wet weights and total soluble protein content were lower in diabetic control group than the rest of experimental groups. Malondialdehyde (MDA)—biomarker of lipid peroxidation- and superoxide dismutase levels had no significant changes between the groups. Catalase (CAT) activities were lower in diabetic groups and the major difference was observed in GR2. Activities of aldose reductase (AR) were higher in diabetic control group and decreased significantly in the treated groups. Also the AR activity was noticeably lower in GR2 than in GR1. Lens enzymatic activities are described in Table 4.

### 2.6. Lens Pathology

**Figure 3:**
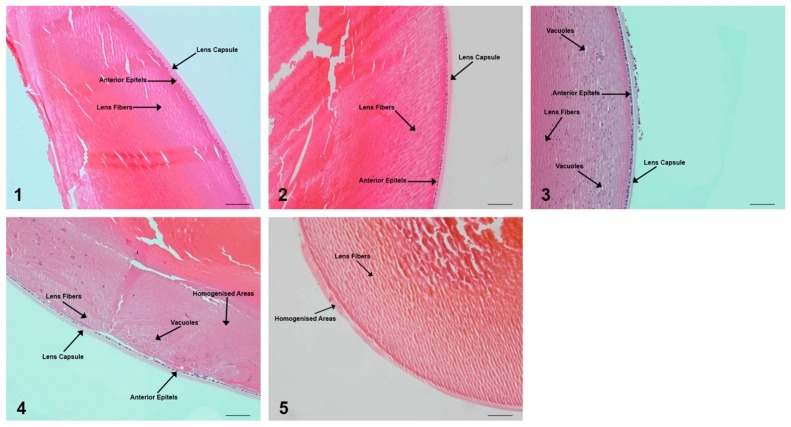
Microphotographs of lens sections stained with hematoxylin and eosin (HEX100). Panels (**1**) and (**2**) indicate the normal lenticular histology of the GR1 and GR2 rats. Panel (**3**) indicates the slight vacuole formation of the GR4 rats. Panel (**4**) demonstrates the progressively increasing appearance of vacuoles and homogenized areas in the GR5 rats. Panel (**5**) shows the loss of the lens capsule and anterior epithelium in the GR3 rats. The scale bar represents 50 µm.

No macroscopic cataract was observed with indirect ophthalmoscopy light during the experimental period. The histopathological grading was developed as follows, and the histopathological profiles are given in Figure 3.

Grade 0: Presence of anterior epithelium with lens fibers and capsule;
Grade 1: Presence of anterior epithelium, lens fibers, vacuoles, and capsule;
Grade 2: Presence of anterior epithelium, lens fibers, vacuoles, homogenized areas, and capsule;
Grade 3: Absence of anterior epithelium, presence of lens fibers, vacuoles, homogenized area;
Grade 4: Presence of lens fibers and homogenized area only.

The rats in GR1 and GR2 were given a histopathological score of 0. The lens capsule and anterior epithelium layers were absent in GR3 rats which resulted in a score 4. The lens fibers, lens capsule, anterior epithelium layer, and slight vacuole (intracellular homogenized pinkish areas) formation were observed in GR4, and it was given a score of 1. Rats in GR5 showed a histopathological score of 2, as the lens capsule and anterior epithelium layers were still intact, the lens fibers were visible in highly homogenized areas, and many vacuole formations were present.

### 2.7. Molecular Docking

**Figure 4:**
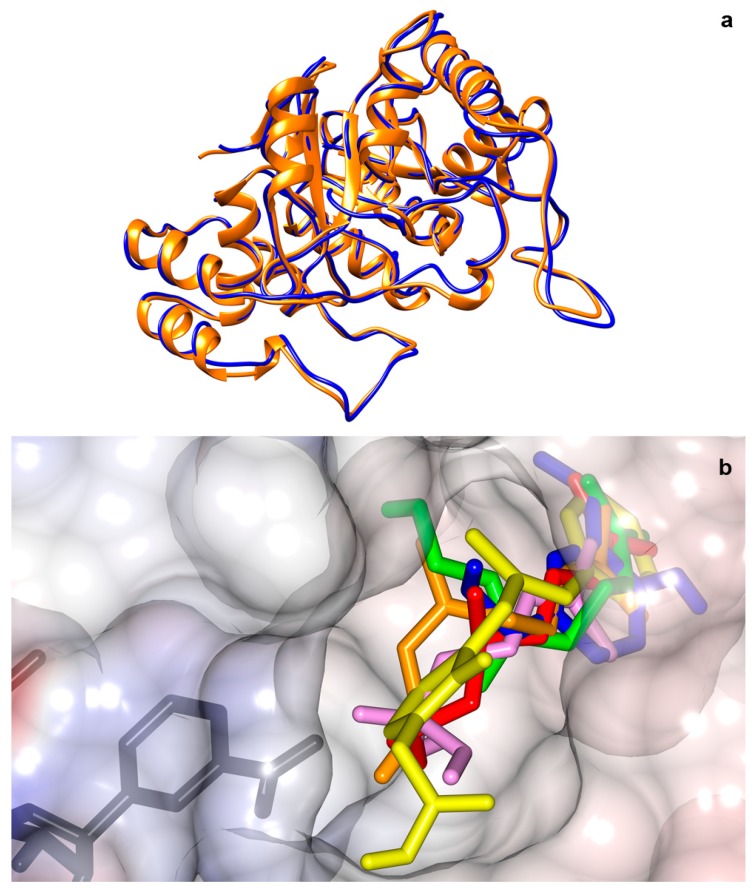
(**a**) Superposition of human (4QXI) and rat (3O3R) aldose reductase (orange and blue, respectively). (**b**) Binding positions of NADP+ (black), epalrestat (yellow), citronellol (red), geraniol (green), nerol (orange), linalool (pink), and eugenol (blue) in human aldose reductase.

As the current study demonstrates, using the effective inhibition of aldose reductase on rats, we investigated the inhibition of aldose reductase with rose hydrosol compounds for humans via molecular docking. Comparison of human aldose reductase to rat aldose reductase resulted in an 80.7% chain of similarity based on the Smith–Waterman algorithm. Docking studies were performed on a crystal structure of human aldose reductase complexed with NADP+ and inhibitor AK198 (4QXI; 0.86 A^0^ resolution) provided by Protein Data Bank. The complexed inhibitor location was selected as the binding side of the protein, then the inhibitor AK198 was removed from the structure. Docking studies were performed using AutoDock Vina script running over UCSF Chimera. The binding scores for epalrestat (positive control aldose reductase inhibitor), citronellol, geraniol, linalool, nerol, and eugenol were generated by AutoDock Vina and were as follows: −9.1 for epalrestat, −6.4 for citronellol, −6.4 for geraniol, −6.5 for linalool, −6.6 for nerol, and −7.3 for eugenol. Superposition of rat and human aldose reductases and binding positions in human aldose reductase are demonstrated in Figure 4.

## 3. Discussion

Diabetes mellitus is one of the most encountered chronic and progressive metabolic disorders characterized by elevated levels of blood glucose due to the fact of insulin resistance or impaired insulin secretion. It also causes oxidative stress affecting all systems via different mechanisms. Streptozotocin is a nitrosourea derivative taken up by pancreatic β-cells which trigger DNA fragmentation over alkylation steps and causes a quick necrosis of β-cells. The rate of insulin synthesis decreases and leads to a stable hyperglycemia [26]. Previous studies indicated that citronellol, geraniol, and eugenol increased insulin-secreting pancreatic cells in diabetic rats and successfully decreased blood glucose levels throughout the treatment period [20,21,22]. In this study, mainly consisting of these molecules, rose hydrosol also regulated the blood glucose levels in treated diabetic rats in a dose-dependent manner.

The STZ-induced diabetic rats showed a significant reduction in body weight owing to the unavailability of glucose as an energy source. We also observed an increase in water intake for the diabetic rats. Excess amounts of glucose are excreted in the urine. Heightened urine secretion leads to dehydration; hence, water intake increases. In comparison with the diabetic control rats, administration of rose hydrosol for 45 days significantly improved weight loss in GR4 and decreased water intake.

Research indicates that alterations in the hematological parameters of humans, such as lower RBC, HGB, and PLT counts and higher WBC counts, occur with DM [27,28,29]. Several studies have reported reference hematological values for healthy rats [30,31,32], and the same alterations were observed in STZ-induced diabetic rats [33,34]. Consistent with previous data, we observed higher WBC counts and lower RBC, HGB, and PLT counts in the diabetic control rats. Treatment with rose hydrosol significantly ameliorated the controlled parameters.

The HbA1c expressed average plasma glucose values over the previous 8 to 12 weeks which is useful in monitoring the effectiveness of treatment [35]. Hemoglobin A1c is formed by the reaction of excess glucose present in blood with HGB [36]. Therefore, HbA1c levels increased in the diabetic rats and the HGB counts were lower. Consuming rose hydrosol over the test period notably decreased the elevated HbA1c levels and increased HGB counts.

High amounts of blood glucose affect the hepatic and renal systems. The AST, ALT, and ALP enzymes are useful biomarkers for determining liver damage. Both human and animal studies indicate that serum ALT, AST, and ALP levels increase with DM [21,22,37,38]. Consistent with previous studies, the diabetic control groups had higher levels of ALT, AST, and ALP. Treatment with rose hydrosol restored hepatic enzymes to a lower level, suggesting that rose hydrosol may have a hepatoprotective effect on hepatic tissue damage caused by STZ injection and induced diabetes. BUN, CREA, and UA are used in the diagnosis of acute or chronic kidney diseases. Urea is the byproduct of protein metabolism and elevated BUN levels are linked to both plasma and liver protein breakdowns. Creatinine is the primary metabolite of creatine which is located in skeletal muscles. Uric acid is also a metabolite, and is the product of purine breakdown. It has previously been reported that BUN, UREA, CREA, and UA levels increase with DM [21,39,40]. The oral administration of rose hydrosol prevented the increase of these markers and has the potential to attenuate renal injury caused by hyperglycemia.

Glycation is the non-enzymatic reaction between macro molecules, such as proteins, lipids or nucleic acids, and reducing sugars. Hyperglycemia is highly associated with increasing protein glycation and accumulation of AGEs in body tissues [41,42,43] which significantly increases the level of inflammation, and it has long been associated with the development of cancer [44]. Plant extracts are already known for their protein glycation inhibiting potentials [45], and eugenol has also been reported as an AGE formation inhibitor [46]. Spectrofluorometric assays revealed that serum AGE levels were significantly lower in rose hydrosol-treated diabetic rats in a dose-dependent manner.

Oxidative stress caused by DM affects the eyes as well. Lens proteins become denatured by glycation and oxidation. Therefore, water soluble protein levels decrease [46]. Malondialdehyde is a marker of polyunsaturated lipid peroxidation in cells. An increase in free radicals causes overproduction of MDA, and it is commonly known as a marker of oxidative stress [47,48]. Superoxide dismutase, CAT, and GPx are antioxidant enzymes which play a fundamental role in antioxidant defense in biological systems against free radicals. Superoxide dismutase catalyzes the dismutation of superoxide anion (O^2-^) into molecular oxygen and hydrogen peroxide. Catalase catalyzes the decomposition of hydrogen peroxide to water and oxygen to prevent oxidation. Glutathione peroxidase reduces soluble hydroperoxides, such as hydrogen peroxide, to water and lipid peroxides to their alcohols [49,50,51,52]. Malondialdehyde levels had no significant differences among groups which indicates that lenses are less prone to lipid peroxidation. The SOD levels also had no prominent differences. Oxidative stress, which triggers the onset and progression of diabetes and its complications, is characterized by a decrease in antioxidants and glutathione. As a result, CAT and GPx levels were lower in the diabetic control group and increased in the treated groups. The highest activity for these two enzymes was observed in GR2. This may be due to the direct enzyme activation or by the reduction of reactive oxygen species.

The polyol pathway is the primary moderator of diabetes-induced oxidative stress in the lens [12]. Under hyperglycemic conditions, AR—the rate limiting enzyme of the polyol pathway—reduces glucose to sorbitol. The hydrophilic nature of sorbitol prevents its extracellular diffusion; hence, it accumulates intracellularly and damages the lens cells [53]. In accordance with the literature, higher AR rates in the diabetic groups were detected. This increase ceased with rose hydrosol therapy. The lowest activity was observed in GR2 which indicates that rose hydrosol components successfully inhibited aldose reductase in all rose hydrosol-consuming groups, even healthy ones.

None of the animals developed macroscopic cataracts during the 45 day experimental period. However, both enzymatic assays and histopathological findings revealed the effects of diabetes on rat lenses and the potential of rose hydrosol in reducing lenticular oxidative stress. The current study demonstrates the effective inhibition of AR in rats; thus, an in silico study is essential in order to extrapolate the results to humans. The comparison of rat and human AR chains led to an 80.70% similarity. The docking results indicated that rose hydrosol components are capable of inhibiting human AR as well.

Statistical analysis showed no significant differences among the non-diabetic groups which indicates that long-term oral consumption of rose hydrosol is non-toxic for the investigated parameters. The only alterations observed were increased CAT and decreased AR activities in GR2 which suggests rose hydrosol as a potent antioxidant for the eyes and an effective AR inhibitor.

## 4. Materials and Methods

### 4.1. Animals and Induction and Diabetes

The 7–8 week old male Sprague–Dawley rats, weighing 220–250 g, were obtained from the Experimental Application and Research Centre of Bezmialem Foundation University (Istanbul, Turkey). Rats were housed in 25 ± 1 °C with a relative humidity of 55 ± 5% on 12 h light–dark cycles. All rats had free access to standard rodent pellets and water ad libitum during the experiments. The experimental procedures were approved by the Laboratory Animals Local Ethics Committee of Bezmialem Foundation University with a protocol number of 2017/203.

Experimental diabetes was induced by a single intraperitoneal injection of 40 mg/kg STZ (Sigma–Aldrich, St. Louis, MO, USA) dissolved in 0.01 M citrate buffer (pH 4.5) in overnight fasted rats. Normal rats received only citrate buffer. Over the next 24 h, the rats were allowed to drink 10% glucose solution to overcome the hypoglycemic effect. Three days after STZ injection, blood samples were collected from tail veins and fasting glucose levels were measured by a blood glucose meter (Accu-Chek Performa Nano, Roche Diagnostics, Mannheim, Germany) with the strips provided. Rats with blood glucose levels above 250 mg/dl were considered as diabetic and were used in the study.

### 4.2. Experimental Protocol

A total of 50 animals were divided into 5 groups. Treatment lasted 45 days, and the experimental groups are described in Table 1. Water bottles were filled with the rose hydrosols and served as the only water source for the experimental groups GR2, GR4, and GR5. Both GR1 and GR3 were given tap water. During the experimental period, water consumption was monitored every day, and the hydrosols were refilled. Body weights were measured only at the beginning and at the end of the experiment. Cataract formation and prevention were monitored at the end of the third and sixth weeks with a handheld indirect ophthalmoscope.

### 4.3. Blood Sampling Procedures for Hematology, Clinical Biochemistry, and AGE Formation

At the end of the experiment, blood samples were collected by cardiac puncture with 22 gauge needles. Samples were transferred to BD Microtainer EDTA tubes (BD Diagnostics, Franklin Lakes, NJ, USA) for hematology and HbA1c analysis. The BD Vacutainer SST II Advance tubes (BD Diagnostics, Franklin Lakes, NJ, USA) were used for biochemistry analysis. Hematology/HbA1c tubes were placed on a roller shaker for a several minutes and immediately analyzed on a Sysmex XS-500i (Hyogo, Japan) analyzer for hematologic parameters. The HbA1c samples were analyzed in a Cobas Integra 400 Plus (Roche Diagnostics, Mannheim, Germany). Samples for biochemical analysis were maintained at room temperature for 15 min then centrifuged at 1500 rpm for 10 min, and the serums were separated. Serum glucose, albumin, bilirubin, globulin, total protein, urea, BUN, uric acid, creatinine, alanine aminotransferase (ALT), aspartate aminotransferase (AST), and alkaline phosphatase (ALP) analyses were also performed on the Cobas Integra 400 Plus with the reagents provided by the manufacturer. Serum AGE concentrations were measured according to a previously published method [54]. Serum samples were diluted 1:50 with 0.01M PBS (pH 7.4) and the fluorescence intensity was recorded at a 440 nm emission upon 350 nm excitation with a Shimadzu RF-5301PC (Shimadzu Scientific Instruments, Kyoto, Japan) spectrofluorometer. Fluorescence intensities were expressed in arbitrary units (RFU; relative florescence unit) proportional to AGE formation.

### 4.4. Collecting the Lenses for Enzymatic Activity and Eyes for Lens Pathology

After collecting the blood samples, the eyes were dissected immediately. Two eyes from different rats per group were fixed in 10% formalin. The paraffin embedded eyes were sectioned at 5 µm thickness and stained with hematoxylin and eosin. The histopathological lens staging was developed with a previously described method [55] with some modifications and carried out by two different pathologists.

The lenses of the remaining eyes were enucleated carefully. Isolated lenses were placed in 2 mL disposable micro homogenization tubes, two lenses per tube in 5 °C cold room temperature. The wet weights of the lenses were recorded and homogenized with 10% (*w*/*v*) 0.01 M PBS (pH 7.4) using a Bio-Gen Pro200 homogenizer (PRO Scientific Inc, Oxford, CT, USA). Homogenates were centrifuged at 10,000× *g* for 1 h. Supernatants were separated and further used for lens enzymatic activities (MDA levels were estimated in homogenates) without any purification. The total soluble protein amounts of the lenses were measured with a micro Lowry total protein assay kit (Sigma–Aldrich, USA). The lipid peroxidation (MDA) assay kit, superoxide dismutase (SOD) determination kit, glutathione peroxidase (GPx) cellular activity assay kit, and catalase (CAT) assay kits were also purchased from Sigma–Aldrich. Aldose reductase—the polyol pathway key enzyme—was measured with a rat aldose reductase (AKR1B1) ELISA kit from Cusabio Technology LLC (Houston, TX, USA). All enzymatic activity assays were performed according to the procedures supplied by the producers with a Bio-Rad xMark microplate reader (Bio-Rad Laboratories, USA).

### 4.5. Statistical Analysis

Statistical analysis was performed using IBM SPSS 22.0 (SPSS Inc., Chicago, IL, USA). Statistical comparisons were made by one-way ANOVA. The Kruskal–Wallis test was used if the dependent variable was not normally distributed. Pairwise comparisons of diabetic groups were achieved with the Bonferroni post-hoc test. Results were articulated as the mean ± SD for ANOVA and the median (minimum–maximum) for the Kruskal–Wallis test. Differences with *p* < 0.05 were considered statistically significant.

## 5. Conclusions

The results of the present study indicate that oral consumption of rose hydrosol has no toxic effect on hematology, renal, and hepatic functions. The treatment of diabetic rats with rose hydrosol resulted in significant alterations in the serum glucose, HbA1c, and AGE levels. It also showed a notable ameliorative effect on hepatic and renal functions in diabetes. By inhibiting the key enzyme of the polyol pathway, rose hydrosol has the potential to be a natural protective agent against diabetic cataracts. However, further studies are essential to ensure the safety and efficacy of rose hydrosol in the extrapolation to diabetes in humans.

## Figures and Tables

**Table 1:** Experimental groups, treatment type, body weight, 45 day average water intake, and 45 day average rose hydrosol volatile consumption.

Group	Body Weight (Start–End) (g ± SD)	Average Water Intake (ml ± SD)	Daily Average Rose Hydrosol Volatiles Intake (mg ± SD)
GR1 (Normal rats supplied with tap water)	236 ± 13–367 ± 34	42 ± 5	None
GR2 (Normal rats supplied with RH1)	236 ± 13–359 ± 42	38 ± 3	57.5 ± 5
GR3 (Diabetic rats supplied tap with water)	236 ± 13–178 ± 27 *	125 ± 12 *	None
GR4 (Diabetic rats supplied with RH1)	236 ± 13–227 ± 12 **	73 ± 23 **	110 ± 35
GR5 (Diabetic rats supplied with RH2)	236 ± 13–208 ± 25	81 ± 12	40.5 ± 6

Total volatile amounts are expressed as citronellol and were calculated with the average water intake value. Data are expressed as the mean ± SD for one-way ANOVA. Significant differences are indicated by * (*p* < 0.05) among all groups and ** (*p* < 0.05) Bonferroni post-hoc test between GR4 and GR3. *n* = 10 per group.

**Table 2:** Results of the hematology analysis.

Parameter	GR1	GR2	GR3	GR4	GR5
WBC (10^3^/µL)	6.26 ± 1.31	7.07 ± 1.23	9.72 ± 1.27 *	7.93 ± 0.59 **	7.99 ± 1.61
RBC (10^6^/µL)	8.11 ± 0.39	8.60 ± 0.46	6.19 ± 0.75 *	8.39 ± 0.42 **	8.54 ± 0.52
HGB (g/dL)	14.43 ± 0.52	14.79 ± 0.67	9.44 ± 0.37 *	14.17 ± 1.37 **	14.86 ± 0.96
HCT (%)	45.84 ± 1.37	46.57 ± 1.92	46.86 ± 2.87	45.73 ± 1.45	46.80 ± 2.24
MCV (fl)	56.58 ± 1.94	56.15 ± 1.98	56.05 ± 1.45	56.00 ± 1.48	55.43 ± 1.16
MCH (pg)	17.80 ± 0.38	17.67 ± 0.52	17.78 ± 0.46	17.68 ± 0.37	17.72 ± 0.35
MCHC (g/dL)	31.55 (30.6–32.3)	31.80 (31.2–32.3)	31.85 (30.2–32.7)	31.80 (30.2–32.1)	32.30 (30.1–33.5)
PLT (10^3^/µL)	760.10 ± 94.74	772.30 ± 88.97	339.70 ± 100.06 *	735.10 ± 92.98 **	670.70 ± 181.14
RDW-SD (fl)	31.31 ± 1.38	30.30 ± 0.92	36.53 ± 2.69 *	30.93 ± 1.28 **	32.43 ± 1.42
RDW-CV (%)	17.99 ± 0.69	18.44 ± 0.69	20.44 ± 1.69 *	18.22 ± 1.03 **	19.37 ± 0.94
PDW (fl)	10.30 (9.4–11.6)	10.86 (10.6–12.7)	11.75 (10.4–21.1)	10.65 (9.4–11.4)	11.45 (9.0–13.4)
MPV (fl)	9.15 (8.6–10.0)	9.60 (9.3–10.6)	9.55 (9.2–10.1)	9.35 (8.6–9.9)	9.55 (9.0–10.2)
P-LCR (%)	18.80 (14.7–25.7)	22.35 (20.2–30.8)	24.05 (18.9–26.9) *	20.40 (14.7–24.6)	22.20 (17.8–28.9)
PCT (%)	0.71 (0.60–0.94)	0.74 (0.60–0.94)	0.77 (0.59–0.83)	0.70 (0.60–0.86)	0.48 (0.30–0.90)
NEUT (%)	41.97 ± 2.78	42.07 ± 3.05	49.52 ± 1.82	42.78 ± 2.41	40.72 ± 2.40
LYMPH (%)	48.18 (46.09–49.86)	47.94 (46.19–49.94)	41.35 (38.53–43.86)	47.30 (46.43–49.92)	47.92 (46.09–48.99)
MONO (%)	8.73 ± 2.34	9.01 ± 2.61	8.67 ± 1.09	8.61 ± 2.20	10.19 ± 2.51
EO (%)	0.90 (0.2–6.40)	1.10 (0.2–1.80)	0.30 (0.10–0.60)	1.30 (0.40–2.20)	0.80 (0.06–6.10)
BASO (%)	0.00 (0.0–1.0)	0.00 (0.0–0.0)	0.00 (0.0–1.0)	0.00 (0.0–0.0)	0.00 (0.0–1.0)

*n* = 10 per group. Data are expressed as the mean ± SD for one-way ANOVA, median (minimum–maximum) for Kruskal–Wallis. Significant differences are indicated by * (*p* < 0.05) among all groups and ** (*p* < 0.05) Bonferroni post-hoc test between GR4 and GR3. (WBC: white blood cell; RBC: red blood cell; HGB: hemoglobin; HCT: hematocrit; MCV: mean corpuscular volume; MCH: mean corpuscular hemoglobin; MCHC: mean corpuscular hemoglobin concentrate; PLT: platelet; RDW-SD: standard deviation of red cell distribution width; RDW-CV: combining red blood cell distribution width; PDW: platelet distribution width; MPV: mean platelet volume; *p*-LCR: platelet larger cell ratio; PCT: plateletcrit; NEUT: neutrophil; LYMPH: lymphocyte; MONO: monocyte; EO: eosinophil; BASO: basophil.)

**Table 3:** The results of the clinical biochemistry analysis.

Parameter	GR1	GR2	GR3	GR4	GR5
GLUC (mg/dL)	129 (115–141)	134 (122–146)	383 (361–434) *	259 (227–289) **	292.5 (276–351)
HbA1c (%)	4.97 (4.11–5.83)	4.73 (4.15–5.31)	11.41 (9.31–13.51) *	8.34 (8.56–9.90) **	9.61 (8.97–10.02)
ALB (g/dL)	3.72 ± 0.25	3.57 ± 0.22	3.48 ± 0.28	3.56 ± 0.42	3.49 ± 0.26
BIL (mg/dL)	0.0460 ± 0.013	0.0440 ± 0.012	0.0540 ± 0.016 *	0.0480 ± 0.001	0.0520 ± 0.013
GLOB (mg/dL)	2.52 ± 0.37	2.66 ± 0.35	3.36 ± 0.40 **	2.87 ± 0.58	3.20 ± 0.61
TP (mg/dL)	6.24 ± 0.41	6.23 ± 0.29	6.85 ± 0.27 *	6.44 ± 0.39 **	6.70 ± 0.45
UREA (mg/dL)	36.00 (28.3–40.6)	35.95 (26.4–38.8)	80.10 (73.1–106.0) *	56.10 (50.8–66.6) **	68.75 (61.3–79.1)
BUN (mg/dL)	16.82 (13.2–18.9)	16.79 (12.8–19.0)	37.43 (34.1–49.5) *	26.21 (23.7–31.2) **	32.12 (28.6–36.9)
UA (mg/dL)	1.01 ± 0.12	1.05 ± 0.18	2.04 ± 0.23 *	1.36 ± 0.11 **	1.53 ± 0.18
CREA (mg/dL)	0.64 ± 0.12	0.67 ± 0.11	2.40 ± 0.21 *	1.50 ± 0.26 **	2.03 ± 0.40
ALT (U/L)	54.74 ± 8.00	53.36 ± 7.38	141.13 ± 16.48 *	86.41 ± 8.62 **	115.17 ± 9.71
ALP (U/L)	131.60 ± 25.41	119.80 ± 15.58	457.80 ± 99.89 *	212.20 ± 40.75 **	333.00 ± 42.49
AST (U/L)	81.83 ± 6.55	80.48 ± 5.92	130.25 ± 8.60*	113.67 ± 3.64 **	121.58 ± 2.79
AGEs (RFU)	13,739.33 ± 434.24	13,201.93 ± 325.08	24,315.31 ± 346.86 *	18,950.85 ± 625.24 **	19,868.20 ± 683.05

*n* = 10 per group. Data are expressed as the mean ± SD for one-way ANOVA, median (minimum–maximum) for Kruskal–Wallis. Significant differences are indicated by * (*p* < 0.05) among groups; ** (*p* < 0.05) Bonferroni post-hoc test between GR4 and GR3.

**Table 4:** Results of the lens enzymatic activities.

Parameter	GR1	GR2	GR3	GR4	GR5
Lens Weight (mg)	31 (30–33)	30 (29–32)	25 (23–28) *	30 (29–32) **	31 (30–32)
Total Lens Protein (mg/100 mg lens)	35.70 (33.10–37.52)	34.55 (33.80–36.85)	28.71 (27.08–30.57) *	35.15 (33.05–37.66) **	36.38 (33.71–37.82)
MDA (µmole/g protein)	2.89 (2.77–2.98)	2.94 (2.80–3.06)	3.01 (2.72–3.09)	2.86 (2.74–2.94)	2.97 (2.78–3.08)
GPx (mU/mg protein)	4.16 ± 0.22	4.59 ± 0.33	2.11 ± 0.19*	4.13 ± 0.64 **	3.67 ± 0.36
SOD (U/mg protein)	37.82 ± 3.16	37.75 ± 3.84	38.89 ± 3.34	39.36 ± 3.53	38.12 ± 2.78
CAT (mU/mg protein)	0.013 (0.009–0.015)	0.019 (0.013–0.021)*	0.010 (0.008–0.12)	0.012 (0.009–0.014)	0.011 (0.008–0.012)
AR (ng/mL)	0.61 ± 0.05	0.49 ± 0.04 ***	1.33 ± 0.32 *	0.62 ± 0.04 **	0.68 ± 0.073

*n* = 9 per group. Data are expressed as mean ± SD for one-way ANOVA, median (minimum–maximum) for Kruskal–Wallis. Significant differences are indicated by * (*p* < 0.05) among groups; ** (*p* < 0.05) Bonferroni post-hoc test between GR4 and GR3; *** (*p* < 0.05) Bonferroni post-hoc test between GR2 and GR1.
